# Influence of HLA-A, -B, -DR Polymorphisms on the Severity of COVID-19: A Case-Control Study in the Iranian Population

**DOI:** 10.34172/aim.2023.40

**Published:** 2023-05-01

**Authors:** Parisa Mashayekhi, Mir Davood Omrani, Zeynab Yassin, Ali Dehghanifard, Leila Ashouri, Sara Sadat Aghabozorg Afjeh, Zahra Shabanzadeh

**Affiliations:** ^1^Molecular Medicine Department, Biotechnology Research Center, Pasteur Institute of Iran, Tehran, Iran; ^2^Urogenital Stem Cell Research Center, Shahid Beheshti University of Medical Sciences, Tehran, Iran; ^3^Antimicrobial resistance Research Center, Institute Of Immunology And Infectious Disease, Iran University of Medical Sciences, Tehran, Iran; ^4^Department of Medical Genetics, Shahid Beheshti University of Medical Sciences, Tehran, Iran

**Keywords:** COVID-19, HLA, Susceptibility

## Abstract

**Background:**

As an emerging pandemic disease, COVID-19 encompasses a spectrum of clinical diagnoses, from the common cold to severe respiratory syndrome. Considering the shreds of evidence demonstrating the relationship between human leukocyte antigen (HLA) allele diversity and infectious disease susceptibility, this study was conducted to determine the association of HLA alleles with COVID-19 severity in Iranian subjects.

**Methods:**

In this case-control study, a total of 200 unrelated individuals (consisting of 100 people with severe COVID-19 and an average age of 55.54 as the case group, and 100 patients with mild COVID-19 with an average age of 48.97 as the control group) were recruited, and HLA typing (Locus A, B, and DR) was performed using the Olerup sequence-specific oligonucleotide (SSO) HLA-typing kit.

**Results:**

Our results showed that HLA-A*11 and HLA-DRB1*14 alleles were more frequently observed in severe COVID-19 cases, while HLA-B*52 was more common in mild cases, which was in agreement with some previous studies.

**Conclusion:**

Our results confirmed the evidence for the association of HLA alleles with COVID-19 outcomes. We found that HLA-A*11 and HLA-DRB1*14 alleles may be susceptibility factors for severe COVID-19, while HLA-B*52 may be a protective factor. These findings provide new insight into the pathogenesis of COVID-19 and help patient management.

## Introduction

 COVID-19, caused by the SARS-CoV-2 virus is considered a pandemic according to the World Health Organization (WHO). This disease primarily affects the lungs. A growing body of evidence demonstrated that a minority of infected individuals developed severe respiratory symptoms and also life-threatening inflammation in other organs.^1^ Several factors have been reported for the variable outcomes of COVID-19, including genetic and environmental factors, pre-existing health conditions, and public health policies.^2,3^ Genetic susceptibility stands out as one of the main risk factors for COVID-19 and now it is established that genetic background plays a critical role in immune responses against the virus and in determining the severity of COVID-19.^4^ Many corresponding studies on COVID-19 and genetic background have identified some genes related to COVID-19 pathogenesis. Although, the risk factors are strong predictors of an increased likelihood of COVID-19 development, there is a paucity of data regarding the role of the main risk factors and COVID-19 severity. Thus, there is an urgent need to determine the association between the genetic susceptibility of subjects and different outcomes of COVID-19.

 Major histocompatibility complex (MHC), a principal part of the immune system, has been highlighted as most closely related to the genetic predisposition to diseases. The human leukocyte antigen (HLA) system is located on chromosome 6 and is the most polymorphic region of the human genome.^5^ The HLA system manages immune regulation; each HLA type recognizes the specific epitopes, then HLA molecules with their attached antigen peptides are recognized by CD8 + or CD4 + T lymphocytes that induce further immunological responses. Therefore, the enhanced binding capabilities of HLA molecules for viral peptides play an essential role in disease outcomes.^6^ So, the more the heterozygosity in HLA alleles, the greater the potential for determining different epitopes, leading to more protection and immunity.^7^ It is thought that the considerable polymorphism in the HLA locus is because of the need for a species to be immunologically diverse to survive a pandemic; therefore, distinct types of HLA are associated with the severity of the viral disease. Previous studies showed the association of HLA diversity with host responses to infectious diseases like HBV, HCV, HIV, SARS-CoV-1,^8-11^ and SARS-CoV-2^12-15^ in different populations. Accordingly, the role of HLA alleles in the immune response to COVID-19 in affected individuals has been of interest to scientists and is under investigation. Considering the shreds of evidence demonstrating the relationship between the diversity of the HLA alleles and susceptibility to infectious diseases, this study was designed to investigate the association of HLA alleles with the severity of COVID-19 in the Iranian population.

## Materials and Methods

###  Sampling and Research Design

 In this case-control study, we included a total of 200 Iranian patients with COVID-19 (100 with mild COVID-19 and 100 with severe COVID-19) with a mean age of 52 (range 22–65 years); 75 were female (mean age 51.14 years) and 125 were male (mean age 52.92 years). Sample collection and subject recruitment were conducted at Rasoul Akram referral hospital of Iran Medical University (Tehran, Iran). All patients had Iranian nationality and also had positive RT-PCR results for SARS-CoV-2. The severe cases were selected from the hospitalized patients requiring admission to the intensive care unit (ICU) between April and October 2021; discharged patients were initially contacted by telephone, and if patients expressed a desire to participate, they were included in the study. Hospital workers with mild COVID-19 were recruited as the mild group. Recruitment was done by reviewing occupational medical records to identify employees who had tested positive on swab tests. They were contacted by phone and invited to participate in the study. Both groups were selected randomly from patients between the ages of 22 and 65. Smokers and patients with chronic comorbidities (cancer, diabetes, hypertension, autoimmune disease, etc) that could have serious or fatal consequences were excluded from the study. Severe and mild COVID-19 were described according to the clinical guidelines from the WHO (https://www.who.int/publications/i/item/clinical-management-of-COVID). The demographic characteristics of the patient group can be seen in Table 1. Two-mL blood samples were collected in EDTA vacutainers from each hospitalized patient who met the WHO guideline for severe COVID-19 and from each mild patient after recovery from the disease.

**Table 1:** Demographic Characteristics of the Participants

**Number (N=200)**	**Severe**	**Mild**
Age (y)	55.54 (30‒65)	48.97 (22‒65)
Male	71	54
Female	29	46

 Molecular analysis was done at Tajrish Research Center, Pasteur Institute of Iran.

###  Diagnosis of SARS-CoV-2 Infection

 Infection with SARS-CoV-2 was confirmed using PowerChek^TM^ SARS-CoV-2 Real-time PCR Kit on StepOnePlus Real-Time PCR Systems (Applied Biosystems, USA) on nasal, nasopharyngeal, and oropharyngeal swab samples from patients with clinical COVID-19 symptoms or a history of close contact with COVID-19 patients.

###  DNA Extraction and HLA Typing

 Blood samples were collected in EDTA vacutainers. Buffy coat was lyzed; then genomic DNA was extracted using the GenAll kit according to the manufacturer’s instructions. A NanoDrop spectrophotometer was used to determine the DNA quantity. To achieve the purity of DNA, the ratio of absorbance at 260/280 nm was used. A ratio of 1.8 to 2.0 was commonly accepted as “pure” for DNA. The ratio of absorbance at 260/230 nm was used as a secondary measure of DNA purity. Approved 260/230 values were in the range of 2.0 to 2.2. HLA typing (Locus A, B, and DR) was performed using the Olerup sequence-specific oligonucleotide (SSO) HLA- typing kit. Then amplified DNA fragments were separated based on their size using agarose gel electrophoresis and interpreted by the SCORE 5 software.

## Statistical Analysis

 We used R (R software; R Foundation for Statistical Computing) package BIGDAWG^16^ for statistical analysis of allele association tests, haplotype estimations, and haplotype association tests; BIGDAWG software was developed to analyze the highly polymorphic HLA data.

 Hardy-Weinberg equilibrium (HWE) tests were calculated using the PyPop software ver. 0.7.0 (http://www.pypop.org). Comparison of allele frequencies in mild and severe groups was carried out using Fisher’s exact test and all statistical values were considered significant at a *P* value of 0.05. The crude odds ratios of each high-risk allele were further calculated, then a multiple logistic regression model was used to adjust for confounding factors (age and sex) and to estimate the adjusted odds ratio and *P*-value.

## Results

###  Distribution of HLA Alleles in Patients with Different COVID-19 Severity

 Demographic data of enrolled individuals were analyzed with respect to disease severity. Results revealed that severe COVID-19 was associated with older age (*P* value < 2.2e–16). Gender was also statistically associated with a more severe course of the disease (*P* value = 0.01)

###  Associations of HLA Alleles with Severe COVID-19 

 We investigated the association between HLA and COVID-19 severity by comparing hospitalized patients against mild patients in the Iranian population.

 The genotype frequencies of HLA-A, -B, and DRB1, did not show any deviation from the HWE (Table 2).

**Table 2:** Hardy-Weinberg Equilibrium

**Locus**	**Patients**			**Control**		
	**Observed Heterozygosity**	**Expected Heterozygosity**	***P *** **Value**	**Observed Heterozygosity**	**Expected Heterozygosity**	***P*** ** Value**
A	0.1752	0.3242	0.0605	0.1377	0.1994	0.1043
B	0.1222	0.1860	0.1378	0.1074	0.0991	0.7334
DRB1	0.1371	0.2091	0.0582	0.1341	0.1994	0.0828

 Analysis of the results demonstrated that A*24, A*02, B*35, B*51, DRB1*11and DRB1*15 were the most prevalent alleles in both case and control groups (Table 3). The comparison of the HLA allele frequencies between the mild and hospitalized patients identified 4 alleles with significantly different frequencies. The alleles A*11 (OR 3.8, 95% CI 1.4‒10.3, *P* = 0.004), B*38 (OR 4.4, 95% CI 0.9–22, *P* = 0.01), and DRB1*14 (OR 2.81, 95% CI 1.09–7.22, *P* = 0.01) presented significant values and were overrepresented in hospitalized COVID-19 patients compared to the mild group which indicated possible predisposing roles for these alleles. The HLA-B*52 allele had a significantly higher frequency in the mild group compared to the hospitalized group (OR 0.2, 95% CI 0.06–0.6, *P* = 0.006); carrying this allele appears to be protective against severe COVID-19.

**Table 3:** HLA Allele Frequencies in Severe and Mild Groups

**Allele**	**Frequency**	
	**Severe**	**Mild**
A*01	10%	11.5%
A*02	15.5%	16.5%
A*03	11.5%	12.5%
A*11	11%	3.5%
A*23	1.5%	2.5%
A*24	19%	20%
A*26	5.5%	5.7%
A*29	3%	2.5%
A*30	7%	6%
A*31	2.5%	2%
A*32	5%	5.5%
A*33	3.5%	4%
A*66	0%	0.25%
A*68	5%	5.5%
B*07	4.5%	4%
B*08	3%	2.5%
B*13	3%	4.5%
B*14	2%	2%
B*15	7%	5.7%
B*18	4.5%	5.5%
B*27	3%	2%
B*35	23%	18%
B*37	1%	3%
B*38	8%	1%
B*40	3.5%	3.5%
B*41	2%	4%
B*44	5%	5%
B*47	0.5%	1%
B*49	4.5%	1.5%
B*50	3%	3%
B*51	10.5%	17.5%
B*52	2%	8.5%
B*53	2.5%	1%
B*55	3%	3.7%
B*57	1.5%	1.5%
B*58	3%	2.5%
DRB1*01	4.5%	2%
DRB1*03	10%	13.5%
DRB1*04	11%	9%
DRB1*07	7%	9%
DRB1*08	3.5%	6%
DRB1*09	0%	0.5%
DRB1*10	2%	2%
DRB1*11	23%	25%
DRB1*12	2.5%	0.5%
DRB1*13	9.5%	11.5%
DRB1*14	12.5%	3%
DRB1*15	13%	14%
DRB1*16	1.5%	4%

 HLA haplotype analysis was carried out and did not show any significant association with the severity of COVID-19 (Table 4).

**Table 4:** Frequencies of Most Represented Haplotypes in COVID-19 Mild and Severe Hospitalized Patients

**A~B~DRB**	**Controls**	**Patients**	**OR**	***P***	**p.c**
24~35~11	0.06	0.04	0.23	0.65	n.s
24~35~04	0.01	0.035	3.591	0.175	n.s
03~35~11	0.015	0.025	1.684	0.724	n.s
03~35~13	0.01	0.02	2.02	0.685	n.s
30~13~07	0.025	0.015	0.594	0.724	n.s
02~51~11	0.02	0.01	0.495	0.685	n.s
01~51~03	0.02	0.005	0.246	0.372	n.s
01~52~15	0.02	0.005	0.246	0.372	n.s

OR, odds ratio; p.c, corrected *P* value; n.s, not significant.

###  Multiple Logistic Regression

 After adjusting for confounding factors, including age and sex, using a multiple logistic regression model, the most robust finding was a significant association between A*11 (OR 3, 95% CI 1.5–9,2, *P*= 0.001) and HLA-DRB1*14 (adjusted OR 2.75, 95% CI 0.98–21.8, *P* = 0.01) with COVID-19 severity, but not for HLA-B*38 (adjusted OR 4, 95% CI 0.98–16,74, *P* = 0.06). Furthermore, the significant protective effect of HLA-B*52 persisted after adjusting for age and sex (adjusted OR 0.18, 95% CI 0.04–0.6, *P* = 0.006) (Table 5).

**Table 5:** HLA Alleles Associated with COVID-19 Severity

**HLA allele**	**OR (95% CI)**	***P *** **Value**	**Adjusted OR (95% CI)**	**Adjusted ** ***P *** **Value**
DRB1*14	2.81(1.09‒7.22)	0.01	2.75 (0.98‒21.8)	0.01
B*38	4.4 (0.9‒22)	0.01	4 (0.98-16‒74)	0.06
A*11	3,8 (1.4‒10.3)	0.004	3.7 (1,5‒9.2)	0.001
B*52	0.2 (0.06‒0.6)	0.006	0.18 (0.04‒0.6)	0.006

OR, odds ratio.

 These results showed that the effect of these alleles on COVID-19 outcomes is independent of patient age and sex.

## Discussion

 A total of 200 unrelated individuals with confirmed COVID-19 diagnoses were enrolled in our study. the case group consisted of 100 people with severe COVID-19 and an average age of 55.54, including 71 men and 29 women; 100 patients with mild COVID-19 were in the control group with an average age of 48.97, including 54 men and 46 women (Table 1). Patients with chronic comorbidities that could lead to severe or fatal outcomes were excluded from the study.

 Confirmed positive cases of COVID-19 show various clinical manifestations from asymptomatic or mildly symptomatic to those who need hospitalization with respiratory support; these various outcomes might be because of several factors that affect the susceptibility or resistance to infectious disease. Notably, host-associated genetic diversity has provided new biological insight into different immune responses against viruses and disease outcomes in affected people. The HLA genes are the most interesting genetic determinators of host-specific immune responses to pathogens. The HLA genes encode glycoproteins that bind pathogenic peptides and display them on the surface of the infected cells to be recognized by specific T lymphocytes for initiating the immune response against the pathogens.^17^

 Although it is well-known that patient HLA profiles play an important role in the development and progression of infectious diseases in general, recently published studies linking HLA alleles and COVID-19 have been largely inconsistent in their findings, which is not necessarily unexpected because of various frequencies of HLA in different populations. In spite of the fact that several studies have illustrated the associations of HLA alleles with COVID-19 infection^18-20^ and some software has been developed to predict the affinity of certain HLAs for viral peptides to assess the association of HLA types with the severity of COVID-19,^14^ some GWA (Genome-Wide Association) studies have reported no association between HLA and COVID-19.^21^

 Given these findings, in this case-control study, we analyzed whether the presence of specific HLA molecules may influence the different outcomes of COVID-19 in a group of Iranian population. Frequency analysis showed that HLA-A*11, HLA-B*38, and HLA-DRB1*14 alleles were significantly associated with severe COVID-19 and HLA-B*52 with mild COVID-19, consistent with some previous studies. This association was no longer significant for HLA-B*38 after age and sex adjustment.

 We found that HLA-A*11 was significantly associated with severe COVID-19. In two earlier studies by Khor et al and Wang et al, HLA-A*11 was enriched in Japanese and Chinese patients with severe COVID-19.^13,22^ Additionally, a cross-sectional study in Greek patients with COVID-19 showed that the frequency of the A*11 allele was higher in hospitalized patients versus mild suffers.^23^ Another study showed that the binding affinity across HLA alleles and viral peptides is weak in patients with HLA-A*11:01 phenotypes, so they are more vulnerable to the severe COVID-19 disease.^14^

 The high frequency of HLA-B*38 in severe COVID-19 in our study was consistent with reports from two previous studies conducted on a limited Iranian population.^24,25^ Furthermore, Shekarkar Azgomi et al investigated the binding affinity of SARS-CoV-2 peptides to MHC class I HLA-A, -B, and –C molecules through an *in silico* method and showed a strong positive correlation of HLA-B*38 with mortality rate.^26^

 In this study, the frequency of HLA-DRB1*14 was significantly higher in severe COVID-19. Another study conducted on 450 Spanish patients hospitalized for COVID-19 found that HLA-DRB1*14 was detected more frequently in the severe COVID-19 group than in the mild group.^19^ Also, Wang et alreported a positive correlation between COVID-19 severity with HLA-DRB1*14 in Chinese patients, but the association was not statistically significant after Bonferroni correction.^22^

 Our result showed that HLA-B*52 frequency was higher in mild COVID-19 patients than in severe patients, implying the protective role of this allele against disease severity; the association persisted after age and sex adjustment. One study assessed HLA frequencies in a cohort of Greek COVID-19 patients and identified a possible negative association of HLA-B*52 in hospitalized patients, suggesting a protective role for this allele against disease severity.^23^ Additionally, Kiyotani et al investigated the possible peptide epitopes that seem to have a high affinity to HLA class I and II molecules to induce the CD8 + and CD4 + T-cell- related immune responses. Their results showed the high binding affinity between SARS-CoV-2 epitopes and the HLA-B*52:01allele.^27^ Although studies on the association between HLA alleles and the severity of COVID-19 have yielded conflicting results, the results of our study suggest that HLA alleles might modify the clinical severity of COVID-19. So, it seems that HLA polymorphism is a determining factor in the outcome of COVID-19 patients.

 In conclusion,the interaction between the virus and the host immune system is the most important determinant of clinical outcomes in people with SARS-CoV-2. Since the HLA system plays a crucial role in activating and regulating the immune response, different HLA types exhibit different behaviors in fighting infection. To illustrate the role of HLA gene polymorphisms in COVID-19 outcomes, in this study, we investigated the association between HLA and COVID-19 severity by comparing hospitalized against mild patients in the Iranian population and found indisputable evidence for the association of HLA alleles with COVID-19 outcomes in Iranians. Our findings may help provide new insight into the pathogenesis of COVID-19 to help develop vaccines and prioritize high-risk individuals for preventive medicine, resulting in reduced morbidity and mortality and a reduced public health burden.

## References

[1] Zaim S, Chong JH, Sankaranarayanan V, Harky A (2020). COVID-19 and multiorgan response. Curr Probl Cardiol.

[2] Minashkin MM, Grigortsevich NY, Kamaeva AS, Barzanova VV, Traspov AA, Godkov MA (2022). The role of genetic factors in the development of acute respiratory viral infection COVID-19: predicting severe course and outcomes. Biomedicines.

[3] Treskova-Schwarzbach M, Haas L, Reda S, Pilic A, Borodova A, Karimi K (2021). Pre-existing health conditions and severe COVID-19 outcomes: an umbrella review approach and meta-analysis of global evidence. BMC Med.

[4] Velavan TP, Pallerla SR, Rüter J, Augustin Y, Kremsner PG, Krishna S (2021). Host genetic factors determining COVID-19 susceptibility and severity. EBioMedicine.

[5] Robinson J, Halliwell JA, McWilliam H, Lopez R, Parham P, Marsh SG (2013). The IMGT/HLA database. Nucleic Acids Res.

[6] Shi Y, Wang Y, Shao C, Huang J, Gan J, Huang X (2020). COVID-19 infection: the perspectives on immune responses. Cell Death Differ.

[7] Sommer S (2005). The importance of immune gene variability (MHC) in evolutionary ecology and conservation. Front Zool.

[8] Sawai H, Nishida N, Khor SS, Honda M, Sugiyama M, Baba N (2018). Genome-wide association study identified new susceptible genetic variants in HLA class I region for hepatitis B virus-related hepatocellular carcinoma. Sci Rep.

[9] Lee MH, Huang YH, Chen HY, Khor SS, Chang YH, Lin YJ (2018). Human leukocyte antigen variants and risk of hepatocellular carcinoma modified by hepatitis C virus genotypes: a genome-wide association study. Hepatology.

[10] Park YJ, Etemad B, Ahmed H, Naranbhai V, Aga E, Bosch RJ (2017). Impact of HLA class I alleles on timing of HIV rebound after antiretroviral treatment interruption. Pathog Immun.

[11] Sun Y, Xi Y. Association Between HLA Gene Polymorphism and the Genetic Susceptibility of SARS Infection. London: IntechOpen; 2014. 10.5772/57561.

[12] Correale P, Mutti L, Pentimalli F, Baglio G, Saladino RE, Sileri P (2020). HLA-B*44 and C*01 prevalence correlates with COVID-19 spreading across Italy. Int J Mol Sci.

[13] Khor SS, Omae Y, Nishida N, Sugiyama M, Kinoshita N, Suzuki T (2021). HLA-A*11:01:01:01, HLA-C*12:02:02:01-HLA-B*52:01:02:02, age and sex are associated with severity of Japanese COVID-19 with respiratory failure. Front Immunol.

[14] Nguyen A, David JK, Maden SK, Wood MA, Weeder BR, Nellore A (2020). Human leukocyte antigen susceptibility map for severe acute respiratory syndrome coronavirus 2. J Virol.

[15] Hajebi R, Ajam A, Karbalai S, Ashraf H, Ostadali Dehaghi MR, Moradi Tabriz H (2021). Association between human leukocyte antigen and COVID-19 severity. Acta Med Iran.

[16] Pappas DJ, Marin W, Hollenbach JA, Mack SJ (2016). Bridging ImmunoGenomic Data Analysis Workflow Gaps (BIGDAWG): an integrated case-control analysis pipeline. Hum Immunol.

[17] Shiina T, Hosomichi K, Inoko H, Kulski JK (2009). The HLA genomic loci map: expression, interaction, diversity and disease. J Hum Genet.

[18] Gutiérrez-Bautista JF, Rodriguez-Nicolas A, Rosales-Castillo A, López-Ruz M, Martín-Casares AM, Fernández-Rubiales A (2022). Study of HLA-A, -B, -C, -DRB1 and -DQB1 polymorphisms in COVID-19 patients. J Microbiol Immunol Infect.

[19] Weiner J, Suwalski P, Holtgrewe M, Rakitko A, Thibeault C, Müller M (2021). Increased risk of severe clinical course of COVID-19 in carriers of HLA-C*04:01. EClinicalMedicine.

[20] Augusto DG, Hollenbach JA (2022). HLA variation and antigen presentation in COVID-19 and SARS-CoV-2 infection. Curr Opin Immunol.

[21] Ellinghaus D, Degenhardt F, Bujanda L, Buti M, Albillos A, Invernizzi P (2020). Genomewide association study of severe COVID-19 with respiratory failure. N Engl J Med.

[22] Wang F, Huang S, Gao R, Zhou Y, Lai C, Li Z (2020). Initial whole-genome sequencing and analysis of the host genetic contribution to COVID-19 severity and susceptibility. Cell Discov.

[23] Detsika MG, Giatra C, Kitsiou V, Jahaj E, Athanassiades T, Kouniaki D (2021). Demographic, clinical and immunogenetic profiles of a Greek cohort of COVID-19 patients. Life (Basel).

[24] Saadati M, Chegni H, Dalir Ghaffari A, Mohammad Hassan Z (2020). the potential association of human leukocyte antigen (HLA)-A and -B with COVID-19 mortality: a neglected risk factor. Iran J Public Health.

[25] Hamidi Farahani R, Esmaeilzadeh E, Nezami Asl A, Heidari MF, Hazrati E (2021). Frequency of HLA alleles in a group of severe COVID-19 Iranian patients. Iran J Public Health.

[26] Shekarkar Azgomi M, Mohammadnezhad L, La Manna MP, Dieli F, Caccamo N (2021). Natural selection footprint in novel coronavirus: a genomic perspective of SARS-COV2 pandemic and hypothesis for peptide-based vaccine. J Biotechnol Biomed.

[27] Kiyotani K, Toyoshima Y, Nemoto K, Nakamura Y (2020). Bioinformatic prediction of potential T cell epitopes for SARS-Cov-2. J Hum Genet.

